# Limitations during Vapor Phase Growth of Bulk (100) 3C-SiC Using 3C-SiC-on-SiC Seeding Stacks

**DOI:** 10.3390/ma12152353

**Published:** 2019-07-24

**Authors:** Philipp Schuh, Johannes Steiner, Francesco La Via, Marco Mauceri, Marcin Zielinski, Peter J. Wellmann

**Affiliations:** 1 Crystal Growth Lab, Materials Department 6 (i-meet), FAU Erlangen-Nuremberg, Martensstr. 7, D-91058 Erlangen, Germany; 2 CNR-IMM, sezione di Catania, Stradale Primosole 50, I-95121 Catania, Italy; 3 LPE S.P.A., Sedicesima Strada, I-95121 Catania, Italy; 4 NOVASiC, Savoie Technolac, BP267, F-73375 Le Bourget-du-Lac Cedex, France

**Keywords:** 3C-SiC, physical vapor transport (PVT), sublimation sandwich, supersaturation, bulk

## Abstract

The growth of 3C-SiC shows technological challenges, such as high supersaturation, a silicon-rich gas phase and a high vertical temperature gradient. We have developed a transfer method creating high-quality 3C-SiC-on-SiC (100) seeding stacks, suitable for use in sublimation “sandwich” epitaxy (SE). This work presents simulation data on the change of supersaturation and the temperature gradient between source and seed for the bulk growth. A series of growth runs on increased source to seed distances was characterized by XRD and Raman spectroscopy. Results show a decrease in quality in terms of single-crystallinity with a decrease in supersaturation. Morphology analysis of as-grown material indicates an increasing protrusion dimension with increasing thickness. This effect limits the achievable maximal thickness. Additional polytype inclusions were observed, which began to occur with low supersaturation (S ≤ 0.06) and prolonged growth (increase of carbon gas-species).

## 1. Introduction

The current knowledge for the growth of silicon carbide using physical vapor transport (PVT) is understood quite well, yielding bulk crystals of up to 200 mm diameter and dislocation densities down to 2800 cm^−2^ [1,2,3]. To date, the consecutive growth and enlargement process for the hexagonal polytypes (4H- and 6H-SiC) using “standard” PVT is not possible for the cubic (3C-SiC) polytype, as a high supersaturation and a temperature gradient is needed to stabilize it. In this context, the sublimation epitaxial approach (SE) using the sublimation sandwich method already presented by Tairov et al. [4] in 1976 was implemented and demonstrated the suitability to grow 3C [5,6]. However, the growth of bulk material using this method is challenging due to the lack of seeding material. Nevertheless, high-quality 3C layers can be obtained using the heteroepitaxial approach of growing the layers on silicon substrates by chemical vapor deposition (CVD) [7,8,9]. Due to the misfit between 3C-SiC and silicon, the wafers are under a lot of stress, leading to bent or even cracked material. By transferring CVD-grown layers on a SiC carrier, a seeding stack can be produced, to perform subsequent growth by SE [10].

Various bulk growth processes for the 3C polytype have been presented over the years, such as the modified PVT (M-PVT) or the continuous-feed PVT (CF-PVT) [6,11,12]. In this work, we will present the growth of 3C-SiC, increasing the thickness using the SE setup and the limitations we faced during the growth on (100)-oriented 3C-SiC-on-Si.

## 2. Experimental Methods

**Figure 1:**
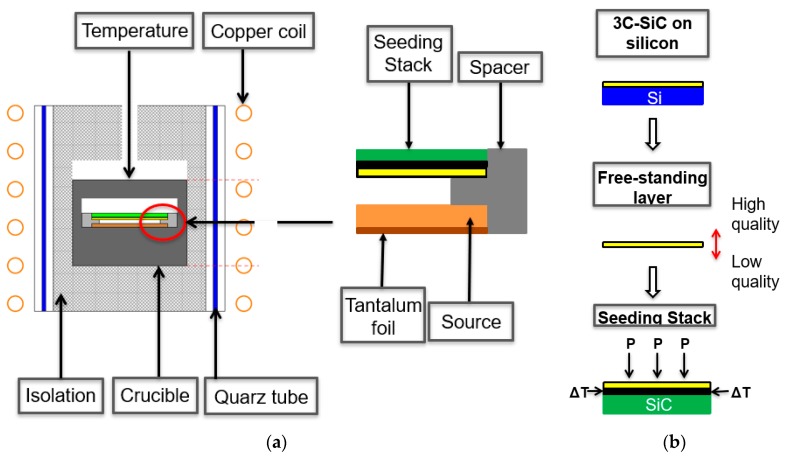
(**a**) Schematic of the used vertical physical vapor transport (PVT) reactor on the left and the hot zone on the right. Starting from the bottom of the hot zone, a tantalum foil is introduced as a carbon getter. On top, a polycrystalline SiC source is placed. The spacer limits the source-to-substrate distance. The final part is the self-manufactured seeding stack. (**b**) The manufacturing process for the seeding stack is necessary for high-temperature growth. The starting material is epitaxial 3C-SiC-on-Si grown by chemical vapor deposition (CVD). The removal of the silicon substrate is performed using wet-chemical etching by HNA (HF:HNO_3_:H_2_O) resulting in a high-quality growth front and a low-quality transition side. The final merging process is performed with a carbon glue layer (black), homogeneous pressure and a specific temperature treatment.

The experimental setup, the calculation of the supersaturation, as well as a description of our transfer process, can be seen in Figure 1. A more detailed description can be found in [10,13]. The transfer of 3C seeding layers was conducted on four-inch wafers, featuring a thickness of approximately 20 µm for the epitaxial 3C layer, on 580 µm thick, highly n-doped, on-axis (100) silicon substrates. We reduced the sample size to 25 × 25 mm^2^ using a diode end-pumped solid-state laser, taking advantage of the multi-pulse ablation effect on 3C-SiC [14,15]. Afterwards, we transferred the obtained layers on 26.5 × 26.5 mm^2^ self-grown, polycrystalline SiC carriers and used the manufactured stacks as seeds for our SE process (Figure 1b) [10]. An additional manual polishing step was added to reduce surface contaminations introduced by the merging process of the carriers and the epitaxial layers. For this purpose, a standard polishing cloth was used, adding a diamond polishing solution with a grain size of ¼ µm.

For the growth of thicker crystals, we increased the source-to-substrate distance from 0.75 mm up to 3 mm. Furthermore, we increased the amount of source material to be similar to the aimed total crystal thickness. A series of samples was grown using different source-to-substrate distances, resulting in thickness values between approximately 450 µm and 650 µm (Figure 1a). An additional experiment was conducted, growing an approximately 2.7 mm thick sample. All experiments were performed in one run, without the exchange of the tantalum getter, located below the source material.

To interpret the results, simulations of the temperature gradient, as well as the supersaturation, were performed using the Multiphysics Software from COMSOL in Stockholm, Sweden. For all computing runs, the backside sublimation was neglected. The altering effect transforming the tantalum getter to tantalum carbide during the process was also taken unaccounted for.

The material was characterized by optical microscopy, Raman spectroscopy and X-ray diffraction using as-grown material. For the morphology and overview images of the samples, we used an optical scanner with incident and through-light setup (HP Scanjet G4050, Palo Alto, CA, USA). The optical images with higher magnification were obtained using a polarization microscope (Axio imager m1m by Zeiss, Oberkochen, Germany). The Raman spectra were obtained using a Horiba (Kyoto, Japan) Jobin Yvon LabRAM HR Evolution confocal microscope (405 nm). The preparation of the cross-cut sample was done using a diamond wire saw and manual polishing with diamond suspension (45 µm–16 µm–15 µm–1 µm).

## 3. Results and Discussion

**Figure 2:**
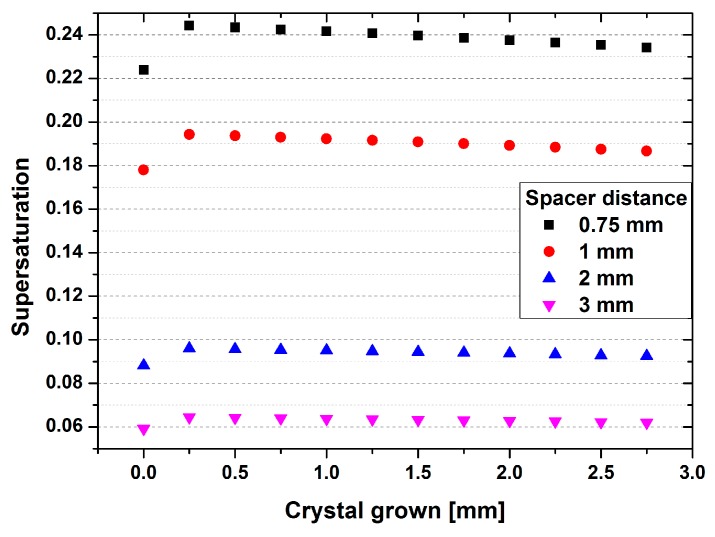
Supersaturation for SiC_2_ gas species as a function of grown crystal thickness. The values were calculated for an overall temperature of 1900 °C for four different source-to-substrate distances.

The nucleation and growth of 3C-SiC is dependent on a high supersaturation, as well as reduced carbon content in the gas phase. The supersaturation is influenced by the temperature gradient between source and seed. The reduction of the carbon content in the gas species is handled by introducing a getter, namely tantalum. This tantalum foil was located below the source material, and also acted as an additional heater. To understand the change of process parameters during the growth, simulations of increasing crystal thickness were carried out. From this data, the temperature gradient was extracted in the middle of a one-inch crystal. The supersaturation was then calculated for the process-limiting gas species SiC_2_. The results are shown in Figure 2.

The plot in Figure 2 shows the variation of supersaturation as a function of grown crystal for four different source-to-substrate distances (0.75, 1, 2 and 3 mm). The simulation was performed in 0.25 mm steps of grown crystal and sublimed source. For all four distances, a slight increase of the supersaturation is visible from 0 mm to 0.25 mm. Afterwards, the numbers slightly decrease with increasing crystal thickness. The main influencing factor for the supersaturation can be assigned to the source-to-seed distance, decreasing from around 0.24 for a distance of 0.75 mm, down to below 0.06 for a distance of 3 mm. Rankl et al. found that the necessary supersaturation for a complete transition from hexagonal SiC to cubic SiC is located close to 0.39 [16]. Using the published growth rate and temperature extracted from that publication, the temperature gradient between source and substrate was simulated, using a new data base for the graphite parts of the growth setup. With these gradients, the partial pressure of the growth-limiting gas species SiC_2_ was calculated following the equations presented in [17,18]. From these numbers, an approximation of the supersaturation can be conducted, decreasing this value to 0.24 for a growth temperature of 1885 °C. This value correlates perfectly with the supersaturation achievable at a source-to-seed distance of 0.75 mm. The growth performed in this work was done on seeding material that had already provided the polytype information of the cubic silicon carbide. The method of transferring epitaxial material on silicon carbide carriers is the first approach of growing homoepitaxial material in a bulk setup. Consequently, the high supersaturation necessary to transform from hexagonal to cubic material presented by Rankl et al. is not needed in this synthesis. Therefore, a reduced supersaturation may lead to a stable growth of 3C-SiC.

**Figure 3:**
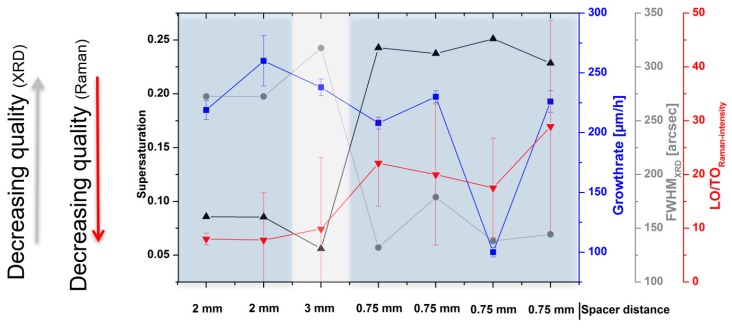
Calculated supersaturation for SiC_2_ gas species for different experiments. Additional information is provided by growth rate (blue) and spacer distance. Analysis data (gray) is extracted from XRD measurements of the 002 peak and the FWHM. Raman data shows the intensity ratio of the LO and the TO mode (red).

Growth runs have been performed for three different source-to-substrate distances (0.75, 2 and 3 mm), resulting in crystals with a thickness between 460 and 640 µm. The samples were characterized using XRD and Raman-spectroscopy. For the analysis of the XRD data, we used the full width at half maximum (FWHM) of the 002 reflex. The Raman results were obtained using the ratio of the intensities of the longitudinal mode (LO) and the transverse optical (TO) mode. The characterization data are shown in Figure 3.

Both characterization methods describe a similar development. With a reduction of the supersaturation, created by an increase in the source-to-substrate distance, the overall material quality decreases. With the values close to 120 arcsec for spacer distances of 0.75 mm, increases of up to 325 arcsec for 3 mm polytype inclusions are highly probable. It is also noteworthy that the growth rate does not seem to influence this property in the used parameter range of the characterized materials.

**Figure 4:**
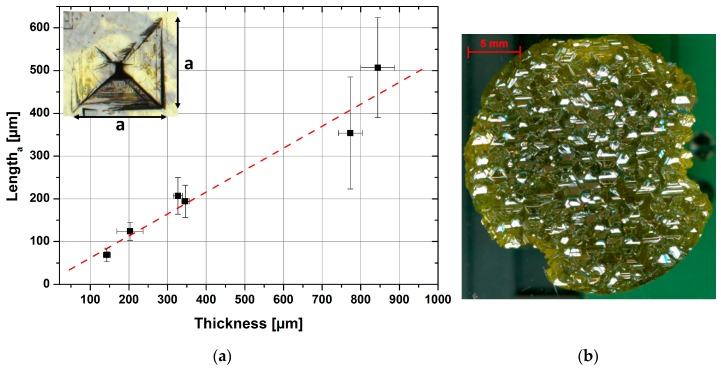
(**a**) Protrusion dimension, measured on the base as a function of crystal thickness. The inset presents a top-down view of a protrusion defect, showing an inverse pyramid. The length a describes the base of such a pyramid created by stacking faults penetrating the surface. (**b**) Surface image of an approximately 2.7 mm thick one-inch sample dominated by the protrusion defect.

The main three-dimensional defect present in cubic silicon carbide grown on silicon is the protrusion. It was already reported by Zimbone et al. [7] that this kind of defect tends to increase in size with increasing thickness of CVD-grown material. This trend can also be observed on bulk material using epitaxial layers as seeds. Figure 4a shows the length of the protrusion base derived from the as-grown surface of samples with increasing thickness. From these numbers, a linear behavior can be extracted. As a consequence, a limitation to the quality of defect-free material is provided by the density of the protrusion defect on the growth front. With a simple extrapolation of the present trend, samples with a thickness of up to 3 mm should feature a protrusion dimension close to 1.4 mm.

A growth run was performed resulting in a sample with a thickness of approximately 2.7 mm. An optical image using incident light is presented in Figure 4b.

As expected, the material surface is dominated by the protrusion defect, increasing the total roughness. Apart from the change in surface morphology, material is absent on the right and bottom-left side. These missing areas were created by backside sublimation during the growth.

**Figure 5:**
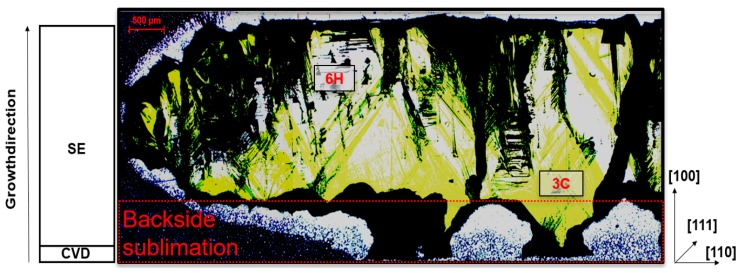
Optical microscope graph in through-light setup of a crosscut, prepared from an approximately 2.7 mm thick one-inch sample. Yellow areas represent cubic silicon carbide. White (transparent) areas describe hexagonal inclusions. Etched areas located at the bottom resemble backside sublimation during the growth.

A cross-cut of this sample is depicted in Figure 5. From this image, the severe backside sublimation can be seen clearly in the bottom areas, stating the necessity of a better backside protection. Apart from the sublimed areas, two distinct areas can be seen in the crosscut. The first one appears in bright yellow and represents cubic silicon carbide. The white/transparent areas can be assigned to 6H-SiC. With an increase in thickness created by prolongation of the growth time a change in growth conditions seems to appear. This change leads to a transition of cubic to hexagonal silicon carbide. However, an additional switch back to cubic also appears to be present as cubic material is present in the top area of the crystal. Therefore, the parameters used for this experiment are located close to the limitations for the cubic polytype, fluctuating during the growth. The black areas in the image can be assigned to cracks, protrusions or certain inclusions.

**Figure 6:**
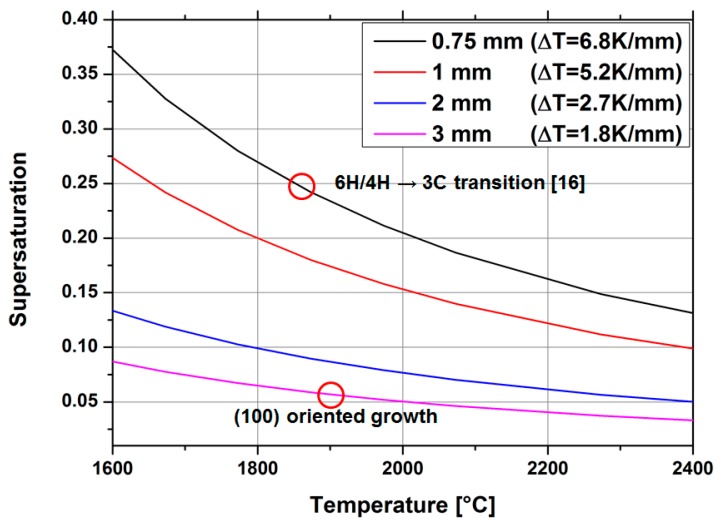
Calculated supersaturation for the growth-limiting gas species SiC_2_ as a function of temperature for four different source-to-substrate distances. Highlighted areas are extracted from the literature and experiments were performed on (100)-oriented material.

From simulation data, the temperature gradient was extracted for four different source-to-substrate distances (0.75, 1, 2 and 3 mm). With these values, the supersaturation was calculated for the growth-limiting gas species SiC_2_ as a function of temperature. The data are presented in Figure 6. The previously discussed supersaturation necessary for a transition from hexagonal to cubic silicon carbide is highlighted with a red circle for a distance of 0.75 mm.

For a source-to-substrate distance of 3 mm, the supersaturation present during the growth of the sample shown in Figure 5 is located below 0.06. As previously mentioned above, a smaller value for the supersaturation may lead to a stable growth of 3C-SiC using this homoepitaxial approach as no transition from hexagonal to cubic is necessary. From the resulting cross-cut and its interpretation, it is reasonable to say that a supersaturation below 0.1 is not beneficial for a stable growth of cubic silicon carbide. This assumption, however, only applies for the use of 3C-SiC-on-SiC seeding material. In this specific case, the polytype information is provided by the interface of the seed and no transition from hexagonal to cubic material is necessary. Additional considerations must be made in regard to the tantalum altering process. With increasing thickness, controlled by either the temperature or the time, the metallic getter will change to tantalum carbide. This process will reduce the gettering, as well as the heating function below the source material.

## 4. Conclusions

A quantitative estimation for the temperature gradient in the sublimation “sandwich” setup was created based on COMSOL Multiphysics. From the acquired data, the supersaturation of the growth-limiting gas species SiC_2_ for different source-to-substrate distances was calculated. Growth of 3C-SiC material in the [100] direction revealed the strong correlation with high supersaturation values.

The main defect limiting the growth with the presented method appears to be the protrusion defect which stems from the 3C-SiC CVD seeding layer. With increasing thickness of the crystal, a linear increasing behavior was observed similar to CVD-grown material. To investigate the material properties further, defect-free seeding layers in terms of protrusions must be used in future work.

Additional comparisons were done with published numbers of the supersaturation for the transition from hexagonal to cubic material. By adapting this information to the herein presented setup and simulation database a corrected value was derived. Experimental results for increased thickness of the material suggest a minimum supersaturation of 0.1 to be sufficient for the growth of cubic silicon carbide.
